# Leveraging Cross-National Environmental Data: Lessons Learned From the Gateway to Global Aging Data

**DOI:** 10.1093/geroni/igaf122.041

**Published:** 2025-12-31

**Authors:** Sara Adar, Emma Nichols

## Abstract

The environments where people live, work, and socialize are known to influence health. However, these relationships can vary across studies, raising questions as to if these inconsistencies stem from differences in populations or variations in study design, data quality, and analytic approaches. Harmonizing data helps to ensure comparability across studies, offering improved insights as to how the environment affects health and why relationships may differ by location. This symposium highlights research within the Gateway to Global Aging Data (Gateway) project, an open access data sharing platform that provides harmonized longitudinal data on the exposome for nine countries around the world. Dr. Adar will first present a summary of the environmental exposome measures available to researchers while Ms. Clements will share the trends in those exposures across places and times. Dr. Ashtan and Zaninotto will then showcase how analyses can be run in individual cohorts showcasing how two measures – greenspace and temperature – are associated with altered mental and cognitive health in India and England. Dr. Zhang will then share an example of leveraging data across studies with a harmonized analysis of air pollution and cognitive function through four HCAP studies. Collectively, these talks will introduce this resource to the research community, share lessons learned from constructing harmonized exposure metrics, and highlight analyses of these exposures on outcomes of aging both within and across countries. We will also discuss how researchers can leverage similarities and differences across locations to advance science and highlight examples of research using Gateway data.

